# Supplementary material for: A Preliminary Study on Factors That Drive Patient Variability in Human Subcutaneous Adipose Tissues
Source: Cells. 2024 Jul 24;13(15):1240. doi: 10.3390/cells13151240 (PMC11311805; doi:10.3390/cells13151240)
Supplement: Supplementary file 1 [file cells-13-01240-s001.zip › cells-3076345-supplementary.pdf]

Supplementary Materials for "A preliminary study on factors that drive patient variability in human subcutaneous adipose tissues"

Megan K DeBari, Elizabeth K Johnston, Jacqueline V Scott, Erica Ilzuka, Wenhuan Sun, Victoria A Webster-Wood, Rosalyn D Abbott

Supplement S1. Statistical Significance for Image Analysis

As would be expected given the diversity of the human samples and how adipocytes vary considerably in size from 20-300 μm [1], there were statistical differences in adipocyte diameters between patients (**Supplemental Figure S1**). The average adipocyte diameter in this study ranged from 55.05 μm (Patient 10) to 88.89 μm (Patient 12) which is within the normal range for human subcutaneous adipose tissue. 6/20 patients were classified as "large" adipocytes as their diameter was on average >70 μm [1]. It is known that adipocyte size affects adipocyte function, where large (70-120 μm) and very large (>120 μm) diameter adipocytes have reduced insulin sensitivity, lower metabolic health, and increased proinflammatory cytokine and free fatty acid secretion [1,2].

The number of adipocytes in each sample was the highest loading in the third dimension of the FAMD (**Table 3**), indicating this was a key driver of variability in human subcutaneous adipose tissues. The correlation matrix indicated that the number of adipocytes decreased with increasing age (-0.55) and as the diameter of the adipocyte increased (-0.47) (**Supplemental Figure S2**). It should be noted that images were taken at the same magnification, therefore large adipocytes would result in fewer cells per frame. However, not all images contained densely packed adipocytes, with many containing collagen-dense areas and extracellular lipid droplets (**Figure 2**). Another interesting finding was the correlation between the number of adipocytes and a longer doubling time of the stromal vascular fraction (**Supplemental Figure S2B**). The more adipocytes there were, the longer it took the stromal vascular fraction to proliferate, indicating hyperplastic growth in the organ limited the cellular proliferative capacity of the remaining preadipocyte population.

The total number of extracellular lipid droplets were counted for each patient (**Supplemental Figure S1J**). Most patients had significantly more extracellular lipid droplets than adipocytes, except for patients 9, 11, 18, and 19. Interestingly, even though there is variability in the number of extracellular lipid droplets to adipocytes, there are no statistically significant differences in the co-localization score of the number of extracellular lipid droplets in collagen/total number of extracellular lipid droplets (**Supplemental Figure S1K**). The co-localization scores are all greater than 0.5, indicating that the majority of extracellular lipid droplets detected were found in collagen-dense areas.

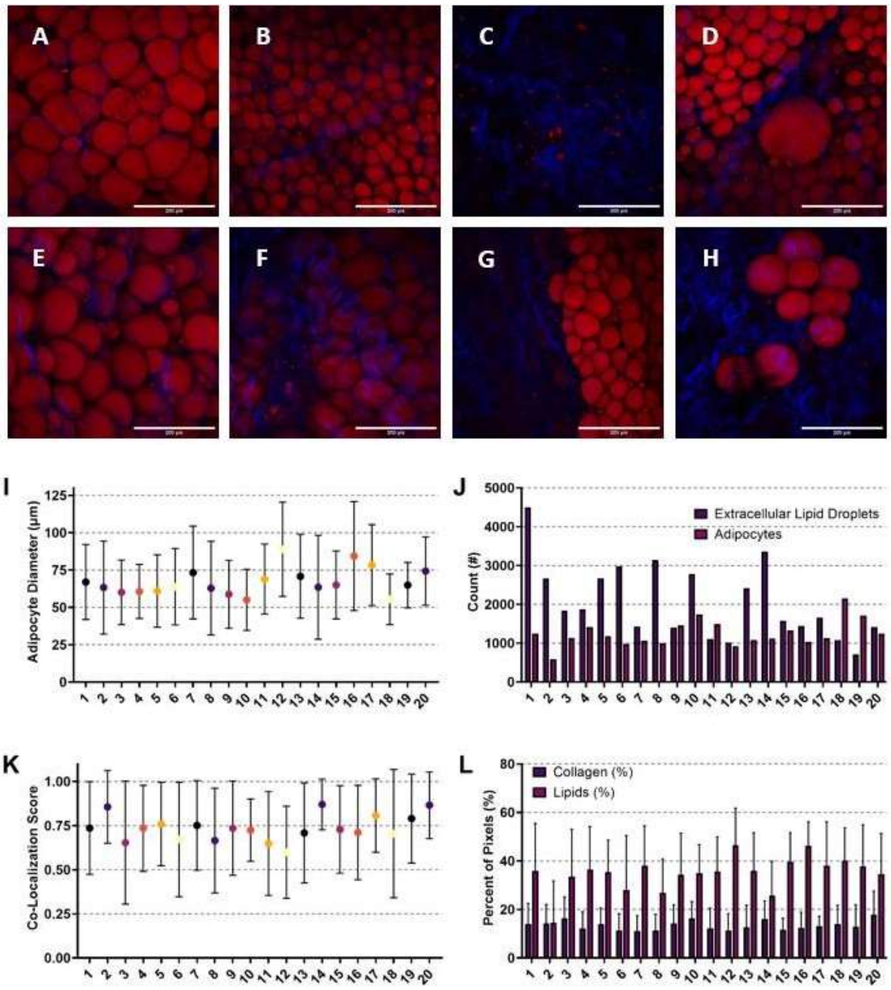

Supplemental Figure S1. While there is considerable variability in size of adipocytes, number of adipocytes, and percentage of lipid/collagen, extracellular lipid droplets are consistently found in collagen-dense regions. Representative images used to analyze number of adipocytes, adipocyte size, extracellular lipid location, and the percentage of lipids and collagen (A-H). Images show lipid droplets (red) and collagen (blue). Scale bars are 200 µm. Each image is from a different patient. Patients had variable adipocyte diameters (I), quantity of extracellular lipid droplets and adipocytes (J), and quantity of collagen (L). However, there was no statistical difference in the co-localization score (K) defined as the number of extracellular lipid droplets in collagen/total number of extracellular lipid droplets. High values for the colocalization score signify more extracellular lipid droplets were found in collagenous regions than in non-collagenous regions of the tissue. This indicates that large variations in the quantity of extracellular lipid droplets and their ratio to adipocytes did not significantly change the location of where the extracellular lipid droplets were distributed (primarily in collagenous regions).

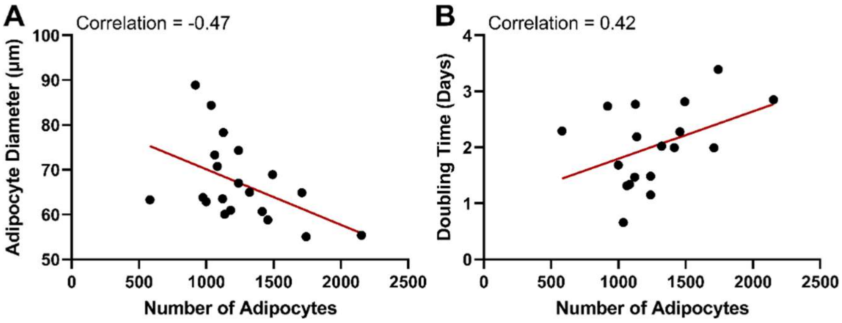

Supplemental Figure S2. **As the number of adipocytes increases the diameter of the remaining adipocytes decreases and the doubling time of the stromal vascular fraction gets longer.** The number of adipocytes in histological images was plotted versus the diameter of adipocytes in the same images (A) and against the recorded doubling time of the stromal vascular fraction seeded into cell culture flasks. Correlations from the correlation matrix are indicated on each plot.

Supplemental Table S1. Statistical significance for adipocyte size. Determined through a one-way ANOVA followed by Tukey's post-hoc analysis. Non-statistically significant comparisons are not shown. Significance was defined as p<0.05. * p<0.05, ** p<0.01, *** p<0.005, **** p<0.0001.

Comparison	Significance	P value
**1 vs. 3**	***	0.0002
**1 vs. 4**	**	0.0026
**1 vs. 5**	**	0.0052
**1 vs. 7**	**	0.0051
**1 vs. 9**	****	<0.0001
**1 vs. 10**	****	<0.0001
**1 vs. 12**	****	<0.0001
**1 vs. 16**	****	<0.0001
**1 vs. 17**	****	<0.0001
**1 vs. 18**	****	<0.0001
**1 vs. 20**	***	0.0005
**2 vs. 7**	****	<0.0001
**2 vs. 10**	***	0.0003
**2 vs. 12**	****	<0.0001
**2 vs. 13**	**	0.0095
**2 vs. 16**	****	<0.0001
**2 vs. 17**	****	<0.0001
**2 vs. 18**	***	0.0004
**2 vs. 20**	****	<0.0001
**3 vs. 7**	****	<0.0001
**3 vs. 11**	****	<0.0001
**3 vs. 12**	****	<0.0001
**3 vs. 13**	****	<0.0001
**3 vs. 16**	****	<0.0001
**3 vs. 17**	****	<0.0001
**3 vs. 20**	****	<0.0001
**4 vs. 7**	****	<0.0001
**4 vs. 10**	*	0.0207
**4 vs. 11**	****	<0.0001
**4 vs. 12**	****	<0.0001
**4 vs. 13**	****	<0.0001
**4 vs. 16**	****	<0.0001
**4 vs. 17**	****	<0.0001
**4 vs. 18**	*	0.0324
**4 vs. 20**	****	<0.0001
**5 vs. 7**	****	<0.0001
**5 vs. 10**	**	0.0089
**5 vs. 11**	****	<0.0001
**5 vs. 12**	****	<0.0001
**5 vs. 13**	****	<0.0001
**5 vs. 16**	****	<0.0001

**5 vs. 17**	****	<0.0001
**5 vs. 18**	*	0.014
**5 vs. 20**	****	<0.0001
**6 vs. 7**	****	<0.0001
**6 vs. 10**	****	<0.0001
**6 vs. 12**	****	<0.0001
**6 vs. 13**	*	0.0135
**6 vs. 16**	****	<0.0001
**6 vs. 17**	****	<0.0001
**6 vs. 18**	****	<0.0001
**6 vs. 20**	****	<0.0001
**7 vs. 8**	****	<0.0001
**7 vs. 9**	****	<0.0001
**7 vs. 10**	****	<0.0001
**7 vs. 12**	****	<0.0001
**7 vs. 14**	****	<0.0001
**7 vs. 15**	****	<0.0001
**7 vs. 16**	****	<0.0001
**7 vs. 18**	****	<0.0001
**7 vs. 19**	****	<0.0001
**8 vs. 10**	***	0.0001
**8 vs. 11**	*	0.0252
**8 vs. 12**	****	<0.0001
**8 vs. 13**	**	0.001
**8 vs. 16**	****	<0.0001
**8 vs. 17**	****	<0.0001
**8 vs. 18**	***	0.0002
**8 vs. 20**	****	<0.0001
**9 vs. 11**	****	<0.0001
**9 vs. 12**	****	<0.0001
**9 vs. 13**	****	<0.0001
**9 vs. 15**	**	0.0041
**9 vs. 16**	****	<0.0001
**9 vs. 17**	****	<0.0001
**9 vs. 19**	**	0.0095
**9 vs. 20**	****	<0.0001
**10 vs. 11**	****	<0.0001
**10 vs. 12**	****	<0.0001
**10 vs. 13**	****	<0.0001
**10 vs. 14**	****	<0.0001
**10 vs. 15**	****	<0.0001
**10 vs. 16**	****	<0.0001
**10 vs. 17**	****	<0.0001
**10 vs. 19**	****	<0.0001
**10 vs. 20**	****	<0.0001
**11 vs. 12**	****	<0.0001
**11 vs. 16**	****	<0.0001
**11 vs. 17**	****	<0.0001
**11 vs. 18**	****	<0.0001
**12 vs. 13**	****	<0.0001
**12 vs. 14**	****	<0.0001
**12 vs. 15**	****	<0.0001
**12 vs. 17**	****	<0.0001
**12 vs. 18**	****	<0.0001
**12 vs. 19**	****	<0.0001
**12 vs. 20**	****	<0.0001
**13 vs. 14**	**	0.0069
**13 vs. 15**	*	0.032
**13 vs. 16**	****	<0.0001
**13 vs. 17**	**	0.0019
**13 vs. 18**	****	<0.0001
**13 vs. 19**	*	0.0414
**14 vs. 16**	****	<0.0001
**14 vs. 17**	****	<0.0001
**14 vs. 18**	****	<0.0001
**14 vs. 20**	****	<0.0001
**15 vs. 16**	****	<0.0001
**15 vs. 17**	****	<0.0001
**15 vs. 18**	****	<0.0001
**15 vs. 20**	****	<0.0001
**16 vs. 17**	*	0.0339
**16 vs. 18**	****	<0.0001
**16 vs. 19**	****	<0.0001
**16 vs. 20**	****	<0.0001
**17 vs. 18**	****	<0.0001
**17 vs. 19**	****	<0.0001
**18 vs. 19**	****	<0.0001
**18 vs. 20**	****	<0.0001
**19 vs. 20**	****	<0.0001

Supplemental Table S2. Statistical significance for collagen and lipid pixel percent. Determined through a two-way ANOVA followed by Tukey's post-hoc analysis. Non-statistically significant comparisons are not shown. Significance was defined as p<0.05. * p<0.05, ** p<0.01, *** p<0.005, **** p<0.0001.

Comparison	Significance	P value
**1: Collagen (%) vs. 1: Lipids (%)**	***	0.0002
**1: Lipids (%) vs. 2: Lipids (%)**	***	0.0003
**2: Lipids (%) vs. 3: Lipids (%)**	**	0.0040
**2: Lipids (%) vs. 4: Lipids (%)**	***	0.0002
**2: Lipids (%) vs. 5: Lipids (%)**	***	0.0005
**2: Lipids (%) vs. 7: Lipids (%)**	****	<0.0001
**2: Lipids (%) vs. 9: Lipids (%)**	**	0.0020
**2: Lipids (%) vs. 10: Lipids (%)**	***	0.0009
**2: Lipids (%) vs. 11: Lipids (%)**	***	0.0004
**2: Lipids (%) vs. 12: Lipids (%)**	****	<0.0001
**2: Lipids (%) vs. 13: Lipids (%)**	***	0.0003
**2: Lipids (%) vs. 15: Lipids (%)**	****	<0.0001
**2: Lipids (%) vs. 16: Lipids (%)**	****	<0.0001
**2: Lipids (%) vs. 17: Lipids (%)**	****	<0.0001
**2: Lipids (%) vs. 18: Lipids (%)**	****	<0.0001
**2: Lipids (%) vs. 19: Lipids (%)**	****	<0.0001
**2: Lipids (%) vs. 20: Lipids (%)**	**	0.0014
**3: Collagen (%) vs. 3: Lipids (%)**	*	0.0234
**4: Collagen (%) vs. 4: Lipids (%)**	****	<0.0001
**5: Collagen (%) vs. 5: Lipids (%)**	***	0.0003
**6: Collagen (%) vs. 6: Lipids (%)**	*	0.0385
**6: Lipids (%) vs. 12: Lipids (%)**	**	0.0062
**6: Lipids (%) vs. 16: Lipids (%)**	**	0.0086
**7: Collagen (%) vs. 7: Lipids (%)**	****	<0.0001
**8: Lipids (%) vs. 12: Lipids (%)**	**	0.0021
**8: Lipids (%) vs. 16: Lipids (%)**	**	0.0030
**9: Collagen (%) vs. 9: Lipids (%)**	**	0.0014
**10: Collagen (%) vs. 10: Lipids (%)**	**	0.0061
**11: Collagen (%) vs. 11: Lipids (%)**	****	<0.0001
**12: Lipids (%) vs. 14: Lipids (%)**	***	0.0005
**14: Lipids (%) vs. 16: Lipids (%)**	***	0.0008
**15: Collagen (%) vs. 15: Lipids (%)**	****	<0.0001
**16: Collagen (%) vs. 16: Lipids (%)**	****	<0.0001
**17: Collagen (%) vs. 17: Lipids (%)**	****	<0.0001
**18: Collagen (%) vs. 18: Lipids (%)**	****	<0.0001
**19: Collagen (%) vs. 19: Lipids (%)**	****	<0.0001
**20: Collagen (%) vs. 20: Lipids (%)**	*	0.0359

Supplement S2. Mechanical properties, DNA content, collagen content, AGEs, metabolic activity, and doubling rate

The elastic modulus, peak stress, stromal vascular fraction (SVF) doubling time, hydroxyproline content, metabolic activity, and advanced glycation end-products (AGEs) content are shown for each patient (**Supplemental Figure S3**). There is large variability between all of the samples for each metric, therefore statistical significance between patients is not shown on graphs and is represented in tabular form (**Supplemental Table S3, Supplemental Table S4, Supplemental Table S5, Supplemental Table S6**).

As indicated on the correlation matrix (**Figure 3**), the highest correlation between quantitative variables in this study (correlation = 0.94) was between the Elastic Modulus and the Peak Stress (**Supplemental Figure S4**). Because these two variables are both gathered from compressive testing, the high correlation is not surprising. Elastic Modulus and Peak Stress define different material behaviors, i.e. the ability of the material to resist elastic deformation ("stiffness" defined as the slope of the stress/strain curve in the elastic region) and the maximum stress (strength) the material withstands, respectively. In many instances, having a high stiffness (Elastic Modulus) can predict the ability to withstand higher stresses. There was also a low correlation of Elastic Modulus and Peak Stress to ACTA2 (0.33 and 0.32, respectfully) and TGFβ1 (0.28 and 0.27, respectfully), which are associated with myofibroblast activation and collagen secretion [3]. Elastic modulus was also a key contributor to tissue variability and contributed the most to the fifth dimension in the FAMD analysis (**Table 3**), demonstrating that tissue mechanics strongly affects other tissue properties.

Both the Elastic Modulus and Peak Stress variables had a low correlation with hydroxyproline content (0.35 and 0.30, respectfully) which follows similar trends, with several exceptions. In general, increased hydroxyproline content, the main component of collagen, was paired with increased elastic moduli and peak stress, with the exception of patient 13 and 15. One reason for these differences between mechanical properties and collagen content is the lack of specificity in the assay. Hydroxyproline assay kits do not differentiate between different types of collagen but simply measure the total hydroxyproline content. Collagens, such as type I, III, IV, V, and VI, are found in adipose tissue and have been linked to obesity and adipose tissue fibrosis [4–7]. However, the role of these collagens varies. Type IV and VI are network-forming collagens found in the basement membrane, while type I, III, and V are fibril-forming collagens found in the interstitial space [8]. Fibrosis can affect the relative concentration of interstitial collagens to basement membrane collagens changing the mechanical properties [9].

To evaluate cellular metabolism, we used resazurin which is transformed from a blue color to the resorufin (pink) by a redox reaction process. Patient 10 had the smallest average adipocyte diameter and the highest metabolic activity while patient 12 had the largest average adipocyte diameter and had a low redox indicator. However, it should be noted that the relationship between adipocyte diameter and what we are defining as "metabolic activity" was not strongly

correlated in our data (-0.22). One study indicated that small adipocytes are insufficient to protect against metabolic dysfunction [10] and that any increase in adipocyte size, either from small or normal size cells, could result in reduced metabolism [1,10]. However, there was a positive correlation between metabolic activity and advanced glycation end products (0.48) supporting literature that shows advanced glycation end products regulate extracellular matrix-adipocyte metabolic crosstalk [11]. Metabolic activity was also negatively correlated with CD86 (M1 macrophage marker, -0.41) indicating adipose tissue metabolism decreased with a proinflammatory M1 macrophage phenotype.

Collectively looking at this dataset, Patient 15 and 16 offer a unique comparison. Both patients are female, age 48, diabetic, non-smoking, with similar BMIs. The only difference between them is that patient 16 has a history of obesity. They do not have statistically different elastic moduli or peak stress but patient 16 has a significantly quicker doubling time and higher metabolic activity. Patient 15 also has a higher hydroxyproline content.

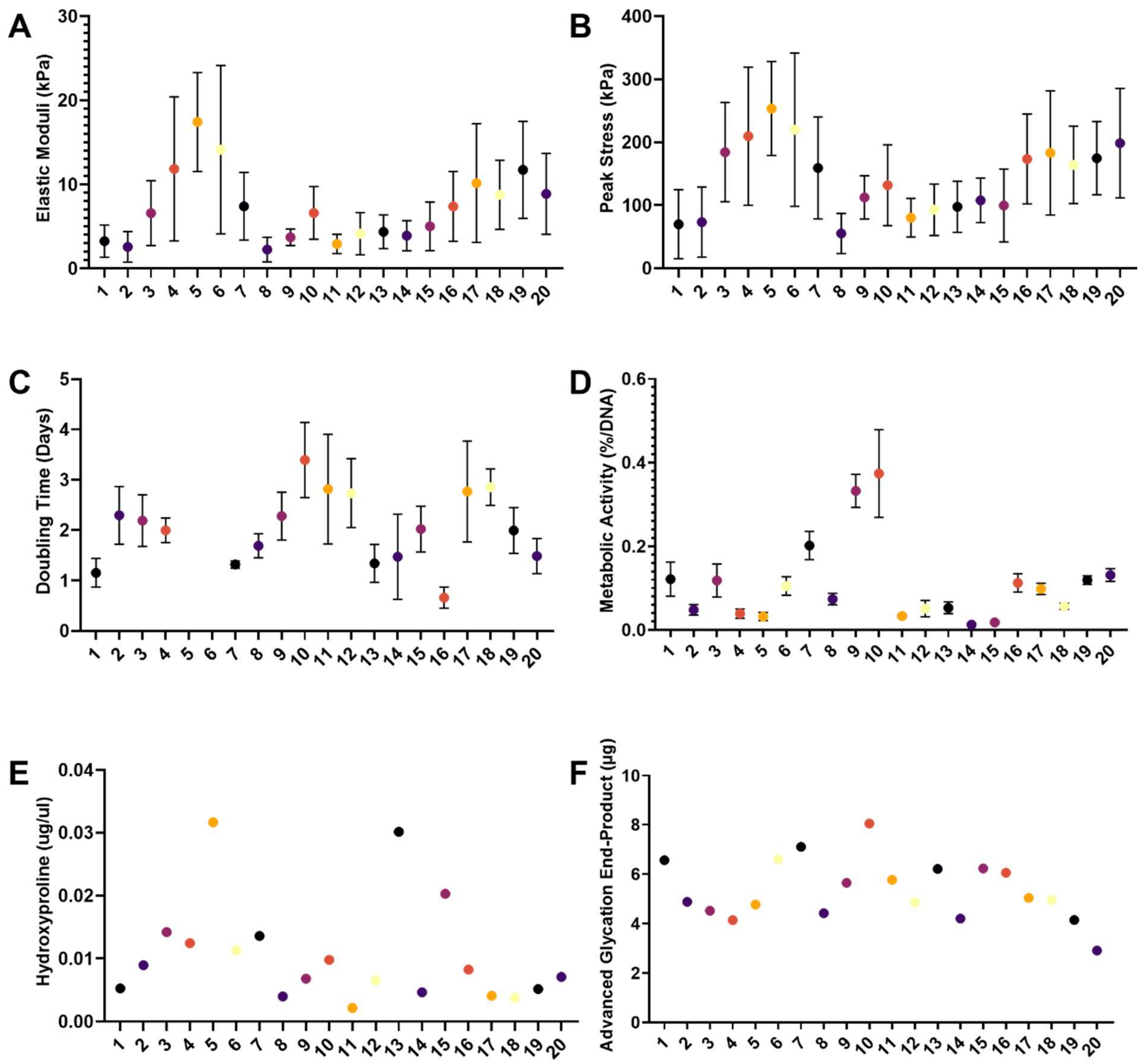

Supplemental Figure S3. (A) Elastic moduli, (B) Peak Stress, (C) SVF Doubling Time, (D) Metabolic Activity, (E) Hydroxyproline, and (F) Advanced glycation end-product concentration for each patient in the study.

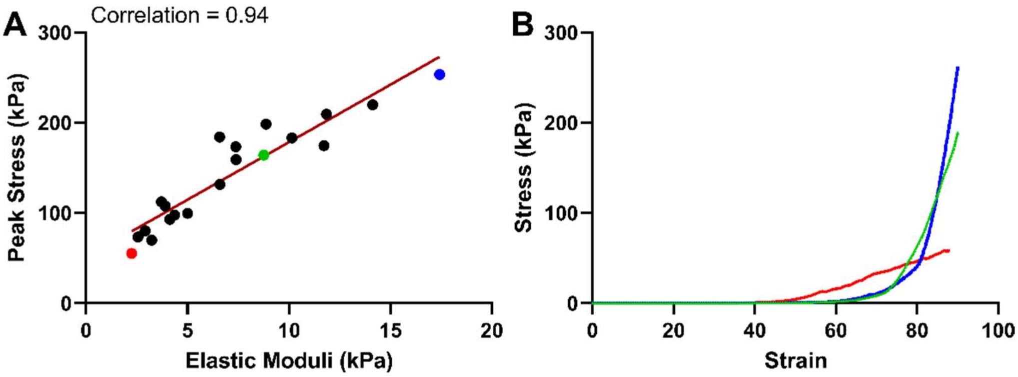

Supplemental Figure S4. (A) Elastic moduli, (B) Peak Stress, (C) SVF Doubling Time, (D) Metabolic Activity, (E) Hydroxyproline, and (F) Advanced glycation end-product concentration for each patient in the study.

Supplemental Table S3. Statistical significance for elastic modulus. Determined through a one- way ANOVA followed by Tukey's post-hoc analysis. Non-statistically significant comparisons are not shown. Significance was defined as $p<0.05$. * $p<0.05$, ** $p<0.01$, *** $p<0.005$, **** $p<0.0001$.

Comparison	Significance	P value
**1 vs. 9**	*	0.025
**1 vs. 11**	**	0.0017
**1 vs. 12**	*	0.0399
**1 vs. 16**	*	0.0251
**1 vs. 17**	**	0.0042
**1 vs. 18**	****	<0.0001
**1 vs. 19**	****	<0.0001
**2 vs. 4**	*	0.0397
**2 vs. 9**	**	0.0011
**2 vs. 11**	****	<0.0001
**2 vs. 12**	**	0.002
**2 vs. 15**	*	0.0112
**2 vs. 16**	**	0.0011
**2 vs. 17**	***	0.0001
**2 vs. 18**	****	<0.0001
**2 vs. 19**	****	<0.0001
**2 vs. 20**	**	0.0038
**3 vs. 19**	*	0.026
**5 vs. 18**	**	0.0029
**5 vs. 19**	***	0.001
**6 vs. 11**	*	0.0105
**6 vs. 17**	*	0.0232
**6 vs. 18**	****	<0.0001
**6 vs. 19**	****	<0.0001
**7 vs. 18**	*	0.0466
**7 vs. 19**	*	0.02
**8 vs. 9**	*	0.0274
**8 vs. 11**	**	0.0019
**8 vs. 12**	*	0.0436
**8 vs. 16**	*	0.0275
**8 vs. 17**	**	0.0047
**8 vs. 18**	****	<0.0001
**8 vs. 19**	****	<0.0001
**10 vs. 18**	*	0.0349
**10 vs. 19**	*	0.0145
**11 vs. 14**	**	0.0053
**13 vs. 18**	**	0.0057
**13 vs. 19**	**	0.002
**14 vs. 17**	*	0.0122
**14 vs. 18**	****	<0.0001
**14 vs. 19**	****	<0.0001

Supplemental Table S4. Statistical significance for peak stress. Determined through a one-way ANOVA followed by Tukey's post-hoc analysis. Non-statistically significant comparisons are not shown. Significance was defined as $p<0.05$. * $p<0.05$, ** $p<0.01$, *** $p<0.005$, **** $p<0.0001$.

Comparison	Significance	P value
**1 vs. 4**	**	0.0091
**1 vs. 5**	****	<0.0001
**1 vs. 6**	**	0.0028
**1 vs. 20**	*	0.0279
**2 vs. 4**	**	0.0028
**2 vs. 5**	****	<0.0001
**2 vs. 6**	***	0.0007
**2 vs. 20**	*	0.0108
**3 vs. 8**	**	0.0048
**4 vs. 8**	***	0.0001
**4 vs. 11**	**	0.0046
**4 vs. 12**	*	0.0282
**4 vs. 13**	*	0.0465
**5 vs. 8**	****	<0.0001
**5 vs. 9**	***	0.001
**5 vs. 10**	*	0.0161
**5 vs. 11**	****	<0.0001
**5 vs. 12**	****	<0.0001
**5 vs. 13**	***	0.0002
**5 vs. 14**	***	0.0008
**5 vs. 15**	***	0.0003
**6 vs. 8**	****	<0.0001
**6 vs. 11**	**	0.0012
**6 vs. 12**	**	0.0087
**6 vs. 13**	*	0.0152
**6 vs. 14**	*	0.0447
**6 vs. 15**	*	0.019
**8 vs. 16**	*	0.0178
**8 vs. 17**	**	0.0055
**8 vs. 18**	*	0.0484
**8 vs. 19**	*	0.0152
**8 vs. 20**	***	0.0007
**11 vs. 20**	*	0.0174

Supplemental Table S5. Statistical significance for SVF doubling time. Determined through a one- way ANOVA followed by Tukey's post-hoc analysis. Non-statistically significant comparisons are not shown. Significance was defined as p<0.05. * p<0.05, ** p<0.01, *** p<0.005, **** p<0.0001.

Comparison	Significance	P value
**1 vs. 10**	****	<0.0001
**1 vs. 11**	**	0.0019
**1 vs. 12**	**	0.0042
**1 vs. 17**	**	0.0031
**1 vs. 18**	**	0.0013
**2 vs. 16**	**	0.0026
**3 vs. 16**	**	0.0069
**4 vs. 10**	*	0.0226
**4 vs. 16**	*	0.0369
**7 vs. 10**	****	<0.0001
**7 vs. 11**	**	0.0093
**7 vs. 12**	*	0.0188
**7 vs. 17**	*	0.0142
**7 vs. 18**	**	0.0066
**8 vs. 10**	**	0.0013
**9 vs. 16**	**	0.0029
**10 vs. 13**	****	<0.0001
**10 vs. 14**	***	0.0001
**10 vs. 15**	*	0.0284
**10 vs. 16**	****	<0.0001
**10 vs. 19**	*	0.0221
**10 vs. 20**	***	0.0002
**11 vs. 13**	*	0.0112
**11 vs. 14**	*	0.0348
**11 vs. 16**	****	<0.0001
**11 vs. 20**	*	0.0388
**12 vs. 13**	*	0.0226
**12 vs. 16**	****	<0.0001
**13 vs. 17**	*	0.017
**13 vs. 18**	**	0.008
**14 vs. 18**	*	0.0255
**15 vs. 16**	*	0.0296
**16 vs. 17**	****	<0.0001
**16 vs. 18**	****	<0.0001
**16 vs. 19**	*	0.0377
**18 vs. 20**	*	0.0286

Supplemental Table S6. Statistical significance for metabolic activity (resazurin). Determined through a one-way ANOVA followed by Tukey's post-hoc analysis. Non-statistically significant comparisons are not shown. Significance was defined as $p<0.05$. * $p<0.05$, ** $p<0.01$, *** $p<0.005$, **** $p<0.0001$.

Comparison	Significance	P value
**1 vs. 2**	*	0.0449
**1 vs. 4**	*	0.0109
**1 vs. 5**	**	0.0034
**1 vs. 7**	*	0.0149
**1 vs. 9**	****	<0.0001
**1 vs. 10**	****	<0.0001
**1 vs. 11**	**	0.0042
**1 vs. 14**	****	<0.0001
**1 vs. 15**	***	0.0002
**2 vs. 7**	****	<0.0001
**2 vs. 9**	****	<0.0001
**2 vs. 10**	****	<0.0001
**2 vs. 20**	**	0.0096
**3 vs. 4**	*	0.0188
**3 vs. 5**	**	0.0061
**3 vs. 7**	**	0.0085
**3 vs. 9**	****	<0.0001
**3 vs. 10**	****	<0.0001
**3 vs. 11**	**	0.0075
**3 vs. 14**	***	0.0002
**3 vs. 15**	***	0.0004
**4 vs. 7**	****	<0.0001
**4 vs. 9**	****	<0.0001
**4 vs. 10**	****	<0.0001
**4 vs. 16**	*	0.0441
**4 vs. 19**	*	0.0156
**4 vs. 20**	**	0.0019
**5 vs. 6**	*	0.0478
**5 vs. 7**	****	<0.0001
**5 vs. 9**	****	<0.0001
**5 vs. 10**	****	<0.0001
**5 vs. 16**	*	0.0156
**5 vs. 19**	**	0.005
**5 vs. 20**	***	0.0005
**6 vs. 7**	***	0.0008
**6 vs. 9**	****	<0.0001
**6 vs. 10**	****	<0.0001
**6 vs. 14**	**	0.0019
**6 vs. 15**	**	0.0049
**7 vs. 8**	****	<0.0001
**7 vs. 9**	****	<0.0001
**7 vs. 10**	****	<0.0001
**7 vs. 11**	****	<0.0001
**7 vs. 12**	****	<0.0001
**7 vs. 13**	****	<0.0001
**7 vs. 14**	****	<0.0001
**7 vs. 15**	****	<0.0001
**7 vs. 16**	**	0.0032
**7 vs. 17**	***	0.0002
**7 vs. 18**	****	<0.0001
**7 vs. 19**	*	0.0103
**8 vs. 9**	****	<0.0001
**8 vs. 10**	****	<0.0001
**9 vs. 11**	****	<0.0001
**9 vs. 12**	****	<0.0001
**9 vs. 13**	****	<0.0001
**9 vs. 14**	****	<0.0001
**9 vs. 15**	****	<0.0001
**9 vs. 16**	****	<0.0001
**9 vs. 17**	****	<0.0001
**9 vs. 18**	****	<0.0001
**9 vs. 19**	****	<0.0001
**9 vs. 20**	****	<0.0001
**10 vs. 11**	****	<0.0001
**10 vs. 12**	****	<0.0001
**10 vs. 13**	****	<0.0001
**10 vs. 14**	****	<0.0001
**10 vs. 15**	****	<0.0001
**10 vs. 16**	****	<0.0001
**10 vs. 17**	****	<0.0001
**10 vs. 18**	****	<0.0001
**10 vs. 19**	****	<0.0001
**10 vs. 20**	****	<0.0001
**11 vs. 16**	*	0.019
**11 vs. 19**	**	0.0062

**11 vs. 20**	***	0.0007
**12 vs. 20**	*	0.0151
**13 vs. 20**	*	0.0206
**14 vs. 16**	***	0.0005
**14 vs. 17**	**	0.0067
**14 vs. 19**	***	0.0001
**14 vs. 20**	****	<0.0001
**15 vs. 16**	**	0.0013
**15 vs. 17**	*	0.0159
**15 vs. 19**	***	0.0004
**15 vs. 20**	****	<0.0001
**18 vs. 20**	*	0.0361

Supplement S3. Gene Expression

SDHA was used as our reference gene due to its high stability in human subcutaneous adipose tissue [12]. Because no control group was used in this study gene expression is expressed as ΔCT values:

$$\Delta CT = CT_{SDHA} - CT_{Target\ gene}$$

Using this formula higher CT values of the target gene, which indicate lower gene expression, would have lower ΔCT values.

Both IL6 and TNFα exhibit higher gene expression in metabolically dysfunctional adipose tissue [5] and were correlated in this dataset (correlation = 0.45). For most patients, a higher IL6 gene expression (**Supplemental Figure S5A**) correlated with a higher TNFα gene expression (**Supplemental Figure S5B**). Interestingly patient 8, the only patient to have an IL6 CT value over 40, also had the lowest TNFα gene expression. We found that both IL6 and TNFα did not correlate with adiponectin, VEGFA, CD86, and CD163 (**Supplemental Figure S6**). TNFα is also linked to increased intracellular lipid accumulation [13]. Interestingly, patients 2, 13, and 14 exhibited increased expression of TNFα expression and also an increased number of extracellular lipid droplets (**Supplemental Figure S1**).

TGFβ1 expression is increased during obesity [14] and when expression is blocked can prevent obesity, insulin resistance, and fatty liver disease [15]. As expected, the patients exhibiting the highest TGFβ1 expression (**Supplemental Figure S5E**) currently have diabetes or are pre- diabetic, which were patients 9, 13 and 16. Older patients, like patients 2, 3, and 17, also experienced an increase in TGFβ1 expression.

Adiponectin inhibits inflammation, which has been shown to reduce collagen content in murine adipose tissue [16,17]. It is exclusively produced by adipocytes. Adiponectin expression is lower in obese and diabetic individuals and increased during weight loss [18]. Due to the nature of the procedure, it is known that the patients that underwent a panniculectomy procedure lost a significant amount of weight, but the time from weight loss is not known. The individuals that have diabetes have a range of gene expression patterns with patients 1 and 8 having decreased gene expression compared to patients 9, 15, 16, and 20 that all experienced increased gene expression (**Supplemental Figure S5I**). Additionally, patient 20 has the highest BMI of the patients we studied.

Leptin has been shown to increase collagen type I content and stimulate TGFβ1 expression [19]. Our results do indicate that patients with higher leptin levels (**Supplemental Figure S5D)** also experienced higher TGFβ1 gene expression (**Supplemental Figure S5C**). However, the leptin levels do not correlate with higher collagen levels. Patients 5 and 13 had the highest hydroxyproline content (**Supplemental Figure S3E**) but have lower leptin gene expression than the majority of other patients.

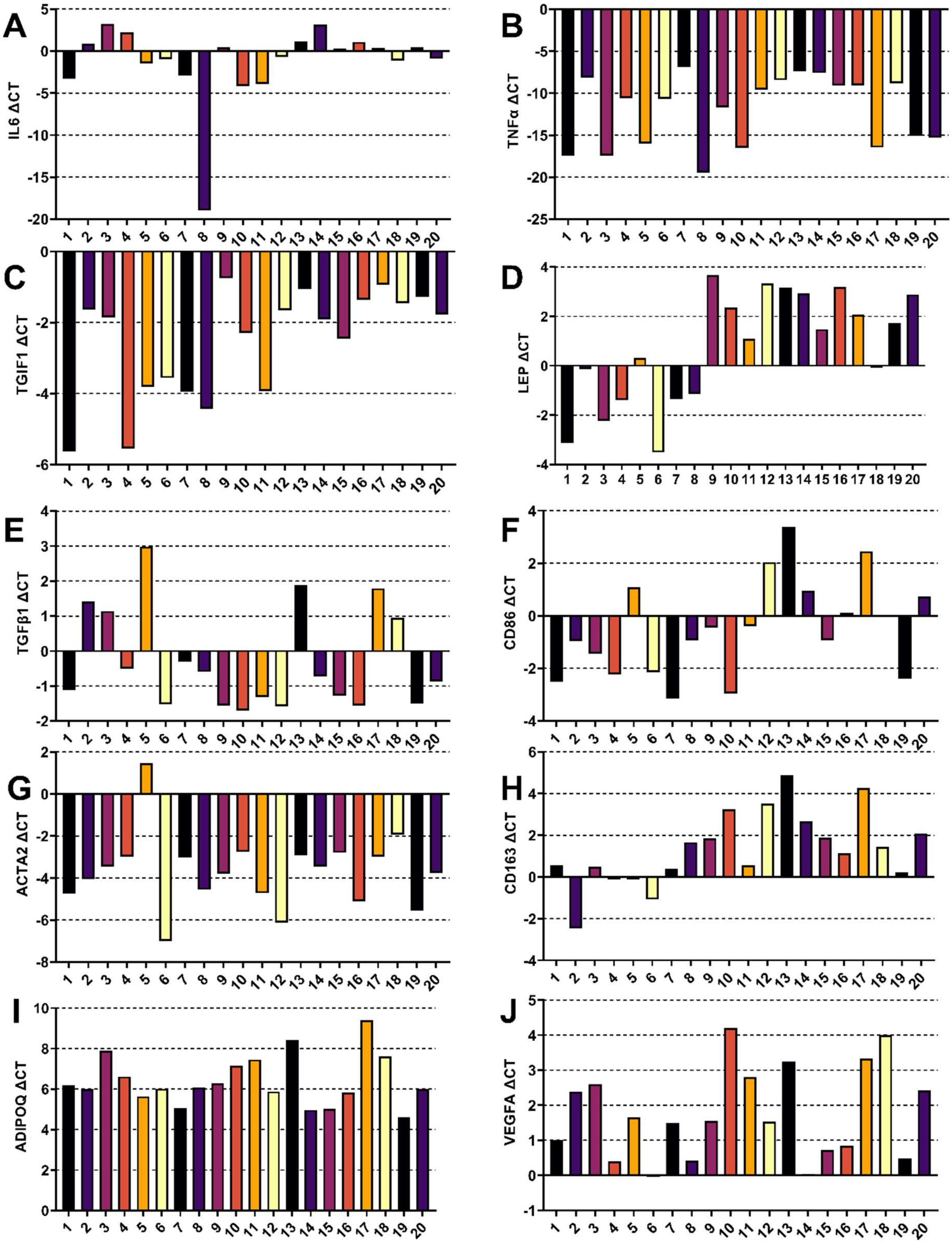

Supplemental Figure S5. Gene expression for each patient shown by the ΔCT value.

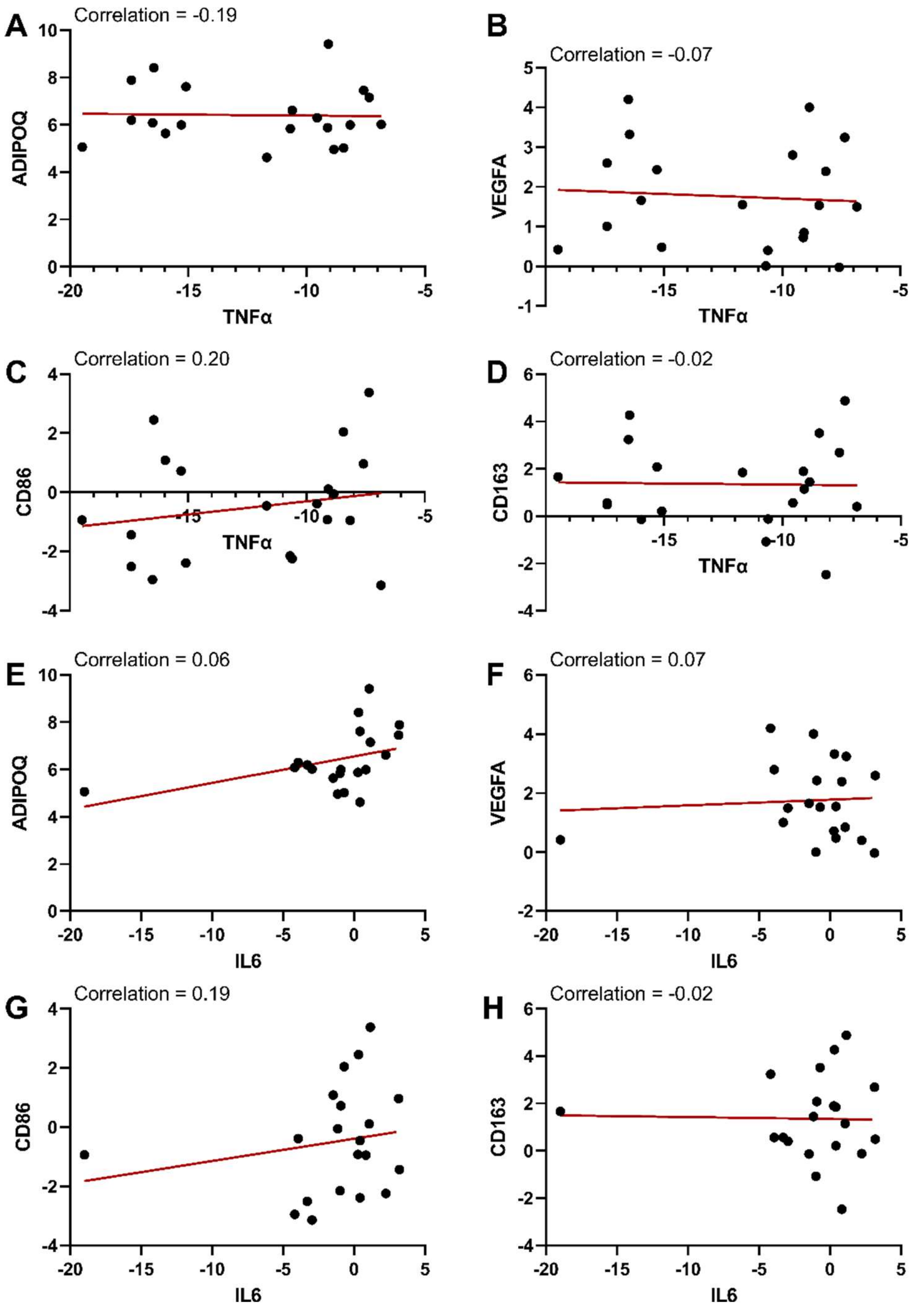

Supplemental Figure S6. Gene expression of inflammatory cytokines (TNF alpha and interleukin 6) were not correlated with adiponectin, VEGFA, CD86, and CD163. TNFalpha (A-D) and interleukin 6 (E-H) were plotted versus adiponectin (A,E), VEGFA (B,F), CD86 (C,G), and CD163 (D,H). Gene expression is represented as a delta CT from the housekeeping gene (ΔCT = CTSDHA – CTTarget gene). With the formula used, gene expression is relative to the housekeeping gene, and increases from a negative value to a higher positive value. Correlations from the correlation matrix are indicated on each plot.

Supplement S4. FAMD coordinates

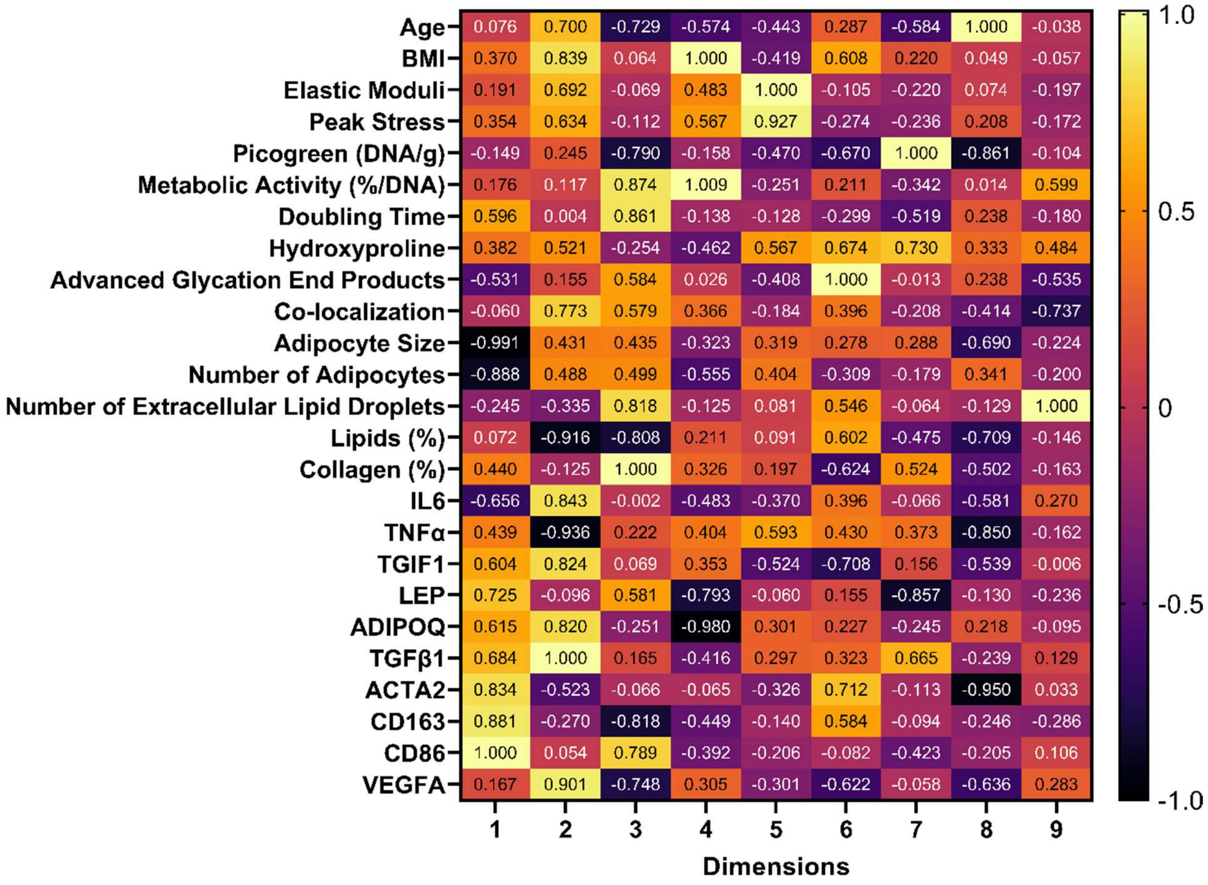

Supplemental Figure S7. Quantitative variable coordinates in each dimension generated from the FAMD analysis normalized to the furthest coordinate in each dimension. High positive coordinate values indicate the variables are clustered and more similar (for example in the first dimension ACTA2, CD86, and CD163), while a negative value would indicate the variables are dissimilar and ordinated further away from that cluster (for example Adipocyte Size and Number of Adipocytes).

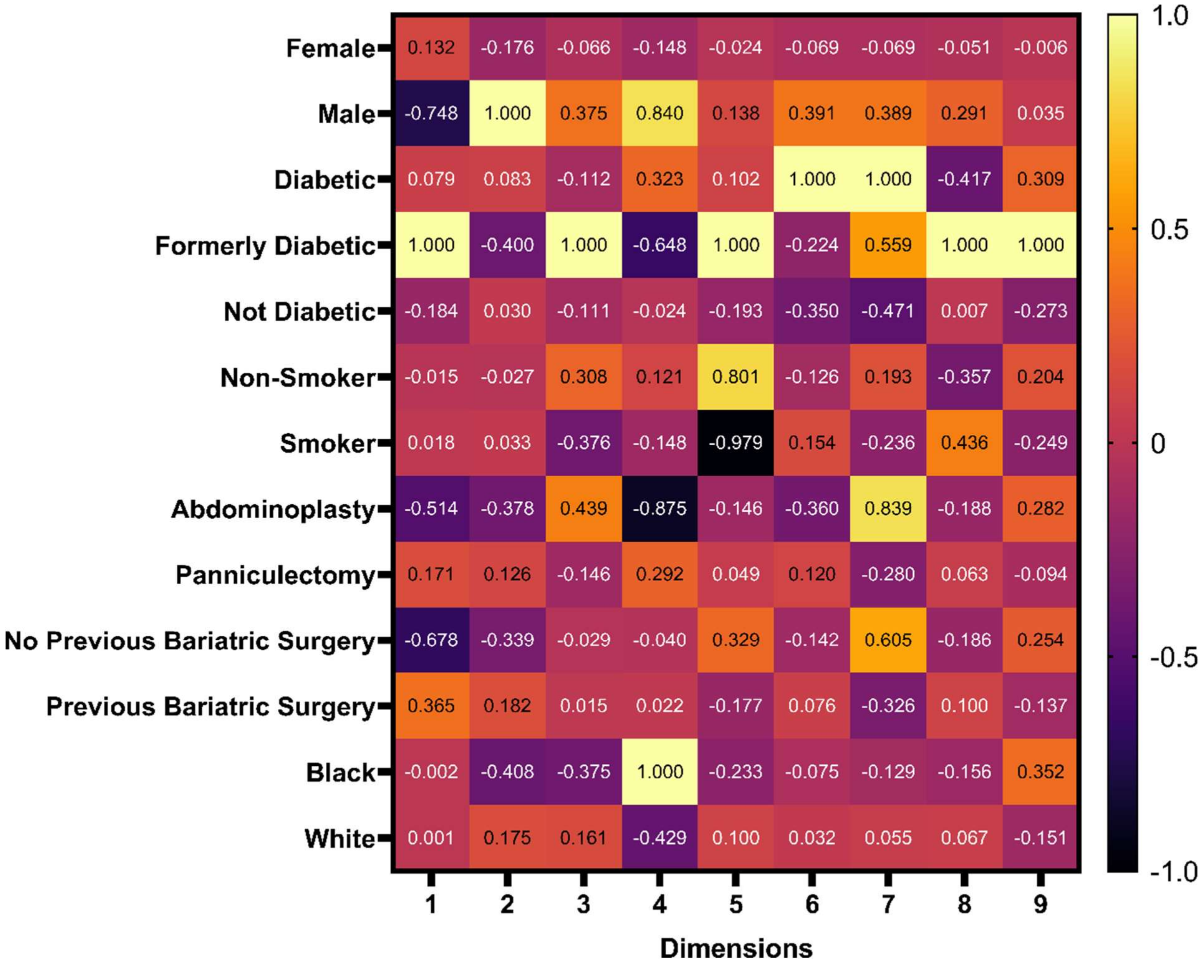

Supplemental Figure S8. Qualitative variable coordinates generated from the FAMD analysis normalized to the furthest coordinate in each dimension.

Supplement S5. Code used for running FAMD

```
library("readxl")
library("ggfortify")
library("tidyverse")
library("gridExtra")
library("scatterplot3d")
library("FactoMineR")
library("ade4")
library("ggrepel")
library("missMDA")
library("factoextra")
library("GPArotation")
library("psych")

#install.packages('writexl')
library(writexl)

#read excel file
my_data.matrix <- read_excel("C:/Users/Megasus/Documents/PCA normalized v4.xlsx")

#store qualitative data as categorical data
factor.race <- as.factor(my_data.matrix$Race)

factor.sex <- as.factor(my_data.matrix$Sex)
my_data.matrix[,2] <- factor.sex
factor.diabetes<- as.factor(my_data.matrix$Diabetes)
my_data.matrix[,3] <- factor.diabetes
factor.smoker<- as.factor(my_data.matrix$Smoker)
my_data.matrix[,4] <- factor.smoker
factor.surgery <- as.factor(my_data.matrix$Surgery)
my_data.matrix[,5] <- factor.surgery
factor.Bariatric.Surgery<- as.factor(my_data.matrix$Bariatric.Surgery)
my_data.matrix[,6] <- factor.Bariatric.Surgery
factor.race <- as.factor(my_data.matrix$Race)
my_data.matrix[,7] <- factor.race


summary(my_data.matrix)

#fill in missing doubling time data points
nb <- estim_ncpPCA(my_data.matrix[1:20,8:32], ncp.min=0, ncp.max=5, method.cv="Kfold")
nb
res.impute <- imputePCA(my_data.matrix[,8:32], ncp=1)
a <- res.impute$completeObs
my_data.matrix[5,14] <- res.impute$completeObs[5,7]
```

```
my_data.matrix[6,14] <- res.impute$completeObs[6,7]

summary(my_data.matrix)

#run FAMD
res.famd <- FAMD(my_data.matrix[1:20,2:32], axes=c(1,2), ncp=32)
summary(res.famd)

# Contribution to the first dimension
fviz_contrib(res.famd, "var", axes = 1, color ="#000000", fill = "#000000" )
# Contribution to the second dimension
fviz_contrib(res.famd, "var", axes = 2, color ="#000000", fill = "#000000")
fviz_contrib(res.famd, "var", axes = 3, color ="#000000", fill = "#000000")
fviz_contrib(res.famd, "var", axes = 4, color ="#000000", fill = "#000000")
fviz_contrib(res.famd, "var", axes = 5, color ="#000000", fill = "#000000")
fviz_contrib(res.famd, "var", axes = 6, color ="#000000", fill = "#000000")
fviz_contrib(res.famd, "var", axes = 7, color ="#000000", fill = "#000000")
fviz_contrib(res.famd, "var", axes = 8, color ="#000000", fill = "#000000")
fviz_contrib(res.famd, "var", axes = 9, color ="#000000", fill = "#000000")

# Display Eigenvalues and variance
eig.val <- get_eigenvalue(res.famd)
head(eig.val)

# Display contribution and coordinates from FAMD analysis
head(res.famd)
```

References:

1. Stenkula, K.G.; Erlanson-Albertsson, C. Adipose cell size: Importance in health and disease. *Am. J. Physiol.-Regul. Integr. Comp. Physiol.* **2018**, *315*, R284–R295
2. Hansson, B.; Moren, B.; Fryklund, C.; Vliex, L.; Wasserstrom, S.; Albinsson, S.; Berger, K.; Stenkula, K.G. Adipose cell size changes are associated with a drastic actin remodeling. *Sci. Rep.* **2019**, *9*, 12941.
3. Kim, K.K.; Sheppard, D.; Chapman, H.A. TGF-β1 Signaling and Tissue Fibrosis. *Cold Spring Harbor Perspect. Biol.* **2018**, *10*, a022293.
4. DeBari, M.K.; Abbott, R.D. Adipose Tissue Fibrosis: Mechanisms, Models, and Importance. *Int. J. Mol. Sci.* **2020**, *21*, 6030.
5. Sun, K.; Tordjman, J.; Clement, K.; Scherer, P.E. Fibrosis and adipose tissue dysfunction. *Cell Metab.* **2013**, *18*, 470–477.
6. Francis, M.P.; Sachs, P.C.; Madurantakam, P.A.; Sell, S.A.; Elmore, L.W.; Bowlin, G.L.; Holt, S.E. Electrospinning adipose tissue-derived extracellular matrix for adipose stem cell culture. *J. Biomed. Mater. Res. Part A.* **2012**, *100*, 1716–1724.
7. Di Caprio, N.; Bellas, E. Collagen Stiffness and Architecture Regulate Fibrotic Gene Expression in Engineered Adipose Tissue. *Adv. Biosyst.* **2020**, *4*, e1900286.
8. Ricard-Blum, S. The collagen family. *Cold Spring Harbor Perspect. Biol.* **2011**, *3*, a004978.
9. Karsdal, M.A.; Nielsen, S.H.; Leeming, D.J.; Langholm, L.L.; Nielsen, M.J.; Manon-Jensen, T.; Siebuhr, A.; Gudmann, N.S.; Ronnow, S.; Sand, J.M.; et al. The good and the bad collagens of fibrosis-Their role in signaling and organ function. *Adv. Drug Deliv. Rev.* **2017**, *121*, 43–56.
10. Johannsen, D.L.; Tchoukalova, Y.; Tam, C.S.; Covington, J.D.; Xie, W.; Schwarz, J.-M.; Bajpeyi, S.; Ravussin, E. Effect of 8 Weeks of Overfeeding on Ectopic Fat Deposition and Insulin Sensitivity: Testing the "Adipose Tissue Expandability" Hypothesis. *Diabetes Care* **2014**, *37*, 2789–2797.
11. Strieder-Barboza, C.; Baker, N.A.; Flesher, C.G.; Karmakar, M.; Neeley, C.K.; Polsinelli, D.; Dimick, J.B.; Finks, J.F.; Ghaferi, A.A.; Varban, O.A.; et al. Advanced glycation end-products regulate extracellular matrix-adipocyte metabolic crosstalk in diabetes. *Scientific Reports* **2019**, *9*, 19748.
12. Perez, L.J.; Rios, L.; Trivedi, P.; D'Souza, K.; Cowie, A.; Nzirorera, C.; Webster, D.; Brunt, K.; Legare, J.F.; Hassan, A.; et al. Validation of optimal reference genes for quantitative real time PCR in muscle and adipose tissue for obesity and diabetes research. *Sci. Rep.* **2017**, *7*, 3612.
13. Maeda, N.; Shimomura, I.; Kishida, K.; Nishizawa, H.; Matsuda, M.; Nagaretani, H.; Furuyama, N.; Kondo, H.; Takahashi, M.; Arita, Y.; et al. Diet-induced insulin resistance in mice lacking adiponectin/ACRP30. *Nature medicine* **2002**, *8*, 731–737.
14. Zeyda, M.; Huber, J.; Prager, G.; Stulnig, T.M. Inflammation correlates with markers of T-cell subsets including regulatory T cells in adipose tissue from obese patients. *Obesity (Silver Spring)* **2011**, *19*, 743–748.
15. Buechler, C.; Krautbauer, S.; Eisinger, K. Adipose tissue fibrosis. *World J. Diabetes* **2015**, *6*, 548–553, doi:10.4239/wjd.v6.i4.548.
16. Khan, T.; Muise, E.S.; Iyengar, P.; Wang, Z.V.; Chandalia, M.; Abate, N.; Zhang, B.B.; Bonaldo, P.; Chua, S.; Scherer, P.E. Metabolic dysregulation and adipose tissue fibrosis: Role of collagen VI. *Mol. Cell. Biol.* **2009**, *29*, 1575–1591.
17. Beltowski, J. Adiponectin and resistin--new hormones of white adipose tissue. *Med. Sci. Monit.* **2003**, *9*, RA55–61.

18. Kern, P.A.; Di Gregorio, G.B.; Lu, T.; Rassouli, N.; Ranganathan, G. Adiponectin Expression from Human Adipose Tissue. *Diabetes* **2003**, *52*, 1779–1785.
19. Wang, J.; Leclercq, I.; Brymora, J.M.; Xu, N.; Ramezani-Moghadam, M.; London, R.M.; Brigstock, D.; George, J. Kupffer cells mediate leptin-induced liver fibrosis. *Gastroenterology* **2009**, *137*, 713–723.
